# Understanding rare and common diseases in the context of human evolution

**DOI:** 10.1186/s13059-016-1093-y

**Published:** 2016-11-07

**Authors:** Lluis Quintana-Murci

**Affiliations:** 1 Human Evolutionary Genetics Unit, Department of Genomes & Genetics, Institut Pasteur, Paris, 75015 France; 2 Centre National de la Recherche Scientifique, URA3012, Paris, 75015 France; 3 Center of Bioinformatics, Biostatistics and Integrative Biology, Institut Pasteur, Paris, 75015 France

## Abstract

The wealth of available genetic information is allowing the reconstruction of human demographic and adaptive history. Demography and purifying selection affect the purge of rare, deleterious mutations from the human population, whereas positive and balancing selection can increase the frequency of advantageous variants, improving survival and reproduction in specific environmental conditions. In this review, I discuss how theoretical and empirical population genetics studies, using both modern and ancient DNA data, are a powerful tool for obtaining new insight into the genetic basis of severe disorders and complex disease phenotypes, rare and common, focusing particularly on infectious disease risk.

## Introduction

Intense research efforts have focused on identifying rare and common variants that increase disease risk in humans, for both rare and common diseases. Several, non-mutually exclusive models have been proposed to explain the functional properties of such variants and their contributions to pathological conditions, and this topic has been reviewed elsewhere [1–10]. These studies implicated multiple variants in disease susceptibility, but the relative importance of rare and common variants in phenotypic diversity, both benign and disease-related, has yet to be explored in detail [11]. We can use an evolutionary approach to tackle this question, as population genetics models can predict the allelic architecture of disease susceptibility [12, 13]. They are able to do so because rare and common disease-risk alleles are a subset of global human genetic diversity, and their occurrence, frequency, and population distribution is governed by evolutionary forces, such as mutation, genetic drift (e.g., migration, admixture, and changes in population size), and natural selection.

**Fig. 1:**
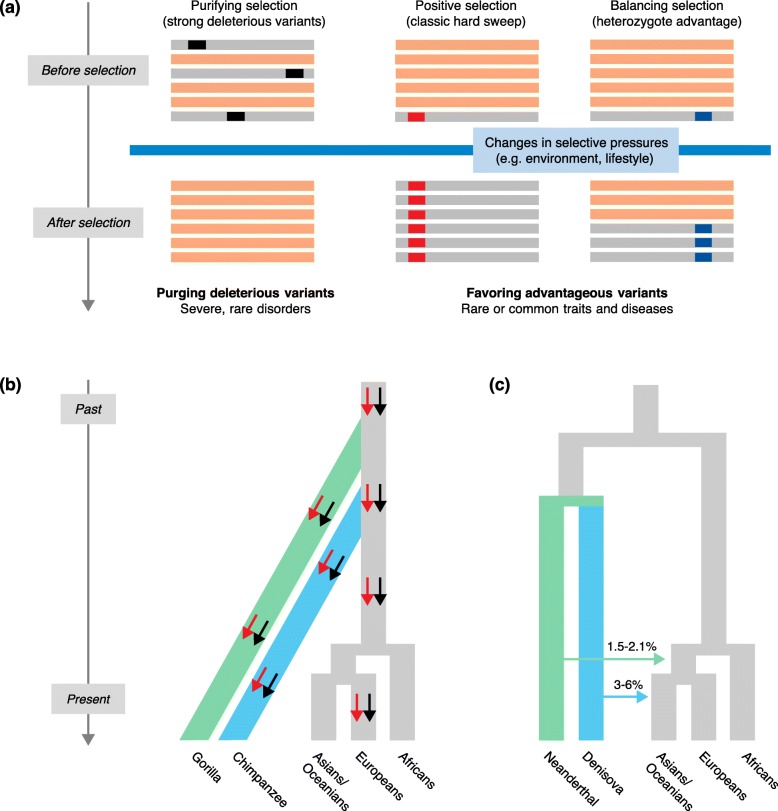
Modes in which selection or admixture can remove, maintain, or increase genetic diversity. **a** Schematic representation of the different types of natural selection. Purifying selection removes deleterious alleles (*in black*) from the population, and genes evolving under strong purifying selection are usually associated with rare, severe disorders. Conversely, mutations conferring a selective advantage (e.g., increased resistance to complex infectious disease) can increase in frequency in the population, or be maintained, through different forms of positive and balancing selection. Positive selection is represented here by the classic hard-sweep model where, following an environmental change, a newly arisen advantageous mutation or a mutation at very low frequency (*in red*) will be immediately targeted by positive selection and will ultimately reach fixation. Balancing selection is illustrated here by the case of heterozygote advantage (or overdominance), where the presence of heterozygotes (*in blue*) is favored in the population. **b** Long-term balancing selection. Advantageous genetic diversity can be maintained over long periods of time and survive speciation, resulting in “trans-species polymorphism” (represented by *black* and *red arrows*). In this example, a trans-species polymorphism that is present in the modern European population (where it has survived the known bottleneck out of Africa) is shared with other primates, such as chimpanzees and gorillas. **c** Modern humans can also acquire genetic diversity (whether advantageous or not) through admixture with other hominins, such as Neanderthals or Denisovans (Box 2). The *green* and *blue arrows* represent the direction and estimated magnitude of admixture between modern humans and Neanderthals and Denisovans, respectively (see [17])

The plethora of genetic information generated in the last ten years, thanks largely to the publication of sequencing datasets for both modern human populations and ancient DNA samples [14–18], is making it possible to reconstruct the genetic history of our species, and to define the parameters characterizing human demographic history: expansion out of Africa, the loss of genetic diversity with increasing distance from Africa (i.e., the “serial founder effect”), demographic expansions over different time scales, and admixture with ancient hominins [16–21]. These studies are also revealing the extent to which selection has acted on the human genome, providing insight into the way in which selection removes deleterious variation and the potential of human populations to adapt to the broad range of climatic, nutritional, and pathogenic environments they have occupied [22–28]. It has thus become essential to dissect the role of selection, in its diverse forms and intensities, in shaping the patterns of population genetic diversity (Fig. 1a), not only to improve our understanding of human evolutionary history, but also to obtain insight into phenotypic diversity and differences in the risk of developing rare and common diseases [12, 13, 24, 29–32].

## The removal of mutations deleterious to human health

Studies of the occurrence, frequency, and population distribution of deleterious mutations are of fundamental importance if we are to understand the genetic architecture of human disease. Theoretical and empirical population genetics studies have shown that most new mutations resulting in amino acid substitutions (non-synonymous) are rapidly culled from the population through purifying selection (Fig. 1a) [33, 34]. Indeed, the small number of non-synonymous variants observed relative to the rate of non-synonymous mutation indicates that most non-synonymous mutations are lethal or highly deleterious, strongly compromising the reproductive success of their carriers [34–36]. Purifying selection—the most common form of selection—refers to the selective removal of alleles that are deleterious, such as those associated with severe Mendelian disorders, or their maintenance at low population frequencies (i.e., mutation–selection balance) [32, 37]. The efficacy of purifying selection for eliminating deleterious mutations from a population depends not only on the selection coefficient(*s*), but also on population size (*N*), which determines the magnitude of genetic drift. Unlike highly deleterious mutations, variants subject to weaker selection (i.e., weakly deleterious mutations) behave like “nearly neutral mutations”; they may, therefore, reach relatively high population frequencies [38–40]. In large outbred populations, with low levels of drift, deleterious mutations will eventually be eliminated. By contrast, in small populations, deleterious mutations behave very much like neutral mutations and may be subject to strong drift, resulting in moderate-to-high frequencies, or even fixation [39].

### Rare variants are widespread in the human genome

Recent deep-sequencing studies are showing a surprisingly high proportion of rare and low-frequency variants in different human populations [14, 15, 41–47]. The Exome Variant Server, for example, reports frequency information from 6515 exomes of individuals of African American and European American ancestry [46]. The most recent release of the 1000 Genomes Project, based on full-genome information for 2504 individuals from 26 populations from around the world, revealed that there was a large number of rare variants in the global dataset (~64 million autosomal variants have a frequency <0.5 %, and only ~8 million have a frequency >5 %), with each individual genome harboring between 40,000 and 200,000 rare variants [15]. A more recent report of high-quality exome data from 60,706 individuals of diverse geographic ancestry, generated as part of the Exome Aggregation Consortium (ExAC), has provided unprecedented resolution for the analysis of low-frequency variants as well as an invaluable resource for the clinical interpretation of genetic variants observed in disease patients [47].

The contribution of rare variants to human disease is a matter of considerable debate, together with the distribution of these variants in the population, as they may underlie early-onset disease and increase susceptibility to common diseases [1, 44, 45, 48–50]. Most rare variants are private to a population, whereas common variants tend to be shared by different populations [51]. Rare variants, particularly those specific to a particular population, tend to have stronger deleterious effects than common variants [42, 52, 53]. Consequently, as shown by population genetics studies, most variants with large functional effects tend to be rare and private, and only a small proportion of variants with large effects are common to different populations. Genome-wide association studies (GWAS), which focus on common variants, have been only moderately successful in explaining the genetic basis of complex diseases [3]. Furthermore, theoretical studies have shown that a large proportion of the so-called “missing heritability” is explained by rare variants, particularly those that affect fitness as well as causing disease [54].

The increasing amount of sequence-based datasets available, both in basic and medically oriented research, is accelerating the investigation into the contribution of rare variants to disease susceptibility. In this context, diverse variant annotation tools and predictive algorithms have been developed to systematically evaluate the potential functional impacts of genetic variants (e.g., PolyPhen, SIFT, and GERP) [55–57], helping to prioritize the study of putative causal variants in further detail. These methods, which use different statistics and types of information, generally assess the “deleteriousness” of each genetic variant by considering different measures, such as evolutionary conservation scores, changes in amino acid sequence, or potential effect on protein function and structure [58]. Novel methods are increasingly being developed, providing improved power and resolution. For example, CADD, which integrates both evolutionary and functional importance, generates a single prediction from multiple annotation sources, including other variant effect predictors [59]. Likewise, MSC provides gene-level and gene-specific phenotypic impact cutoff values to improve the use of existing variant-level methods [60].

Quantification of the burden of deleterious, mostly rare, variants across human populations and an understanding of the ways in which this burden has been shaped by demographic history are now key issues in medical research, because they could help to optimize population sampling and, ultimately, to identify disease risk variants.

### Expansion out of Africa and the patterns of rare, deleterious variants

The sizes of human populations have changed radically over the last 100,000 years, due to range expansions, bottlenecks, and rapid growth over different timescales [18–21]. Several studies have evaluated the impact of such demographic events on the distribution of deleterious variants and have shown that populations that have experienced bottlenecks, such as non-Africans, have higher proportions of deleterious variants of essential genes than African populations. This pattern has been interpreted as resulting from weaker purifying selection due to the Out-of-Africa bottleneck [45, 52, 61]. Nevertheless, an absolute increase in the number of rare functional variants has been observed in populations of African and European descent, relative to neutral expectations, due to the combined effects of an explosive expansion over recent millennia and weak purifying selection [41–46]. Furthermore, ~85 % of known deleterious variants appear to have arisen during the last 5000 to 10,000 years, and these variants are enriched in mutations with a (relatively) large effect as there has not yet been sufficient time for selection to eliminate them from the population [46]. Furthermore, deleterious mutations in Europeans appear to have occurred after those in Africans (~3000 vs. 6200 years ago, respectively) [46], highlighting the effects of demographic history on the distribution of deleterious variants within the population.

**Fig. 2:**
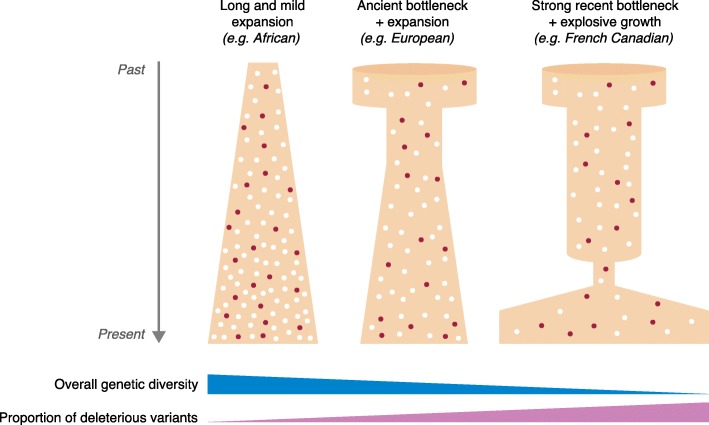
Demographic history affects the proportion of deleterious variants in the human population. The proportion of deleterious variants currently segregating in the population can vary depending on the past demographic regime of each population. Under a regime of demographic expansions alone, populations display higher levels of genetic diversity (in total absolute counts) and lower proportions of deleterious variants (in *brown*) than under regimes in which populations have experienced bottlenecks or recent founder events, where the opposite patterns are observed. The schematic demographic models presented here illustrate the broad demographic history of some modern human populations (e.g., Africans, Europeans, and French Canadians), but they do not attempt to capture their precise changes in population size over time

However, some studies have suggested that demographic history may have a less straightforward impact on the mean burden of deleterious variants [62–64]. Simons and coworkers concluded that individual mutation load is insensitive to recent population history [64], and Do and coworkers suggested that selection is equally effective across human populations [62]. Several factors underlie these apparently conflicting conclusions, including differences in the choice of statistics and the features of genetic variation used to assess the burden of deleterious variation, and differences in the choice of predictive algorithms for defining deleteriousness, together with differences in the interpretations of the results; these factors have been reviewed in detail elsewhere [22, 65]. Nevertheless, all these studies converge to suggest that demographic history affects deleterious and neutral variants differently (Fig. 2), and that mutation and drift have stronger effects on the frequency of weakly deleterious mutations in bottlenecked populations than in large, expanding populations.

### Founder effects and bottlenecks increase the burden of deleterious variation

Besides the impact of long-term population demographics (i.e., African vs. non-African populations) on the distribution of deleterious variants, a few studies have evaluated the effects of more recent, or stronger, changes in population demography. For example, it has been shown that French Canadians have both lower levels of diversity and a larger proportion of deleterious variants than the present-day French population. These findings highlight how a recent major change in population demographics (i.e., a small founder population of ~8500 French settlers subsequently growing by about 700-fold to attain its present size) can profoundly affect the population’s genetic landscape within as little as 400 years [66]. Likewise, the Finnish population, which experienced a recent population bottleneck estimated to have occurred ~4000 years ago, has larger proportions of rare deleterious alleles, including loss-of-function variants and complete gene knockouts, than other populations in Europe or of European descent [67].

Henn and coworkers investigated the consequences of a serial founder effect model for the distribution of deleterious mutations using a set of African populations and several groups located at different geographic distances from Africa [68]. Using explicit demographic models and considering different selection coefficients and dominance parameters, they found that non-African individuals carried larger proportions of deleterious alleles, mostly of modest effect, than African individuals, and that the number of homozygous deleterious genotypes carried by individuals increased with distance from Africa [68]. These results highlight the interaction between drift and purifying selection by showing that deleterious alleles previously maintained at low frequencies by purifying selection may have surfed to higher frequencies in populations at the edge of the wave expanding out of Africa, due to stronger drift [53, 68, 69]. Together, these studies suggest that demographic history has played a central role in shaping differences in the genetic architecture of disease between human populations through its effects on the frequency of deleterious alleles [64, 70].

## Favoring advantageous variants to increase adaptation

Besides the interplay between drift and selection to remove deleterious mutations, other *de novo* or already existing variants can be advantageous and can increase in population frequency through various forms of positive and balancing selection [23–28, 71, 72]. Humans occupy diverse habitats and have gone through many different cultural and technological transitions; human populations have had to adapt to such shifts in habitat and mode of subsistence [25]. Dissecting the legacy of past genetic adaptation is thus key to identifying the regions of the genome underlying the broad morphological and physiological diversity observed across populations, and to increasing our understanding of the genetic architecture of adaptive phenotypes in health and disease.

### Positive selection targets mendelian and complex traits

Positive selection can manifest in different guises: from the classic, hard-sweep model, in which a new mutation can confer an immediate fitness benefit (Fig. 1a), to alternative models of genetic adaptation, such as selection on standing variation or polygenic adaptation [73, 74], with each type of selection leaving a specific molecular signature in the targeted region (reviewed in [23, 26]). Most studies have focused on signals of positive selection according to the hard-sweep model, providing insight into the nature of adaptive phenotypes (see [23, 24, 26, 29, 31, 72, 75–77] and references therein). These phenotypes range from Mendelian traits (or almost so)—including the largely supported lactase persistence trait in various populations [78–82] and traits relating to infectious disease resistance (e.g.,*G6PD*, *DARC*, *FUT2*) in particular (reviewed in [76])—to complex traits, such as skin pigmentation [83–86], adaptation to climate variables or high altitude [87–93], and the immune response and host–pathogen interactions [24, 29, 31, 77, 94–107]. These examples reveal the potent selective pressures that have been exerted by nutritional resources, climatic conditions, and infectious agents since humans first began to spread over the globe [29, 31, 72, 77, 96, 108].

Many selection signals were detected by candidate-gene approaches, based on *a priori* choices of the genes and functions to be investigated. However, a large number of genome-wide scans for positive selection have identified several hundred genomic regions displaying selection signals, consistent with the likely presence in these regions of beneficial, functional variants [28, 37, 109–124]. For example, Grossman and coworkers identified about 400 candidate regions subject to selection, using whole-genome sequencing data from the 1000 Genomes Project [28]. These regions either contain genes involved in skin pigmentation, metabolism and infectious disease resistance, or overlap with elements involved in regulatory functions, such as long intergenic noncoding RNAs and expression quantitative trait loci (eQTL). The presence of non-synonymous variants in less than 10 % of the candidate-selected regions suggests that regulatory variation has played a predominant role in recent human adaptation and phenotypic variation [28], as previously suggested [125–128].

The large number of studies searching for selection signals contrasts with the much smaller number of studies trying to determine when selection effects occurred [83, 129, 130]. Nevertheless, such studies could identify specific time periods corresponding to abrupt changes in environmental pressures. Studies aiming to date the lactase persistence allele in Europe have suggested that this allele was selected in farmers some 6000 to 11,000 years ago [79, 81, 95, 129, 130], although estimates based on ancient DNA point to a more recent time [131, 132] (see below). A recent study, using an approximate Bayesian computation framework, found that skin pigmentation alleles were generally much older than alleles involved in autoimmune disease risk, whose ages are consistent with selection during the spread of agriculture [129]. A report suggesting that many selective events targeting innate immunity genes have occurred in the last 6000 to 13,000 years [95] provides additional support for the notion that the adoption of agriculture and animal domestication modified human exposure to pathogens, leading to genetic adaptations of immune response functions.

Selection studies have thus increased our knowledge of the nature of several adaptive phenotypes at different timescales (Box 1), but the relative importance of selection according to the classic sweep model remains unclear. Several studies have reported the prevalence of classic sweeps for human adaptation to be non-negligible [28, 109–113, 115–118, 122], whereas others have suggested that such sweeps are rare and that the corresponding signals probably result from background selection [74, 93, 123, 124]. There is also increasing evidence to suggest that other, largely undetected forms of genetic adaptation, such as selection on standing variation, polygenic adaptation, and adaptive introgression [73, 74], may have occurred more frequently in the course of human evolution than previously thought (see for example [108, 130, 133–135]).

### Maintaining diversity through balancing selection

Balancing selection can preserve functional diversity, through heterozygote advantage (or overdominance; Fig. 1a), frequency-dependent selection, advantageous diversity fluctuating over time and space in specific populations or species, and pleiotropy [27, 136, 137]. Unlike other forms of selection, balancing selection can maintain functional diversity over periods of millions of years because selection conditions remain constant over time and are strong enough to avoid the loss of selected polymorphisms due to drift. In some cases, polymorphisms subject to balancing selection can persist during speciation events, resulting in trans-species polymorphism (long-term balancing selection; Fig. 1b). In other cases, balancing selection may occur only in particular species or populations, owing to specific environmental pressures (see [27, 136] and references therein). Until a few years ago, evidence for the action of balancing selection was restricted to a few loci, including the sickle cell hemoglobin polymorphism (HbS), which protects against malaria in the heterozygous state [138], and several genes of the major histocompatibility complex (MHC, or HLA in humans), which presents intracellular peptides to cells involved in immune surveillance and triggers immune responses against diverse pathogens [139–141].

Recent studies, bolstered by the whole-genome sequence data published for humans and other species, have suggested that balancing selection is more prevalent than previously thought (see [27] for a review). Several studies searching for the occurrence of trans-species polymorphism have shown that advantageous variants in the human population may have been inherited from distant ancestral species [142–145]. For example, functional diversity in ABO blood group has been maintained across primates for millions of years, probably due to host–pathogen coevolution [142]. Likewise, a scan of long-term balancing selection in the genomes of humans and chimpanzees has detected 125 regions containing trans-species polymorphisms, principally in genes involved in immune function, such as *IGFBP7* and membrane glycoprotein genes; these findings suggest that there has long been functional variation in response to pressures exerted by pathogens in these species [144]. Other studies have searched for balancing selection within humans through the use of genome-wide approaches or by focusing on particular gene families. Selection signatures have been detected in multiple regions, including the *KIR* gene regions (*KIR* genes are known to co-evolve with their HLA ligands [146]), and regions encoding various molecules involved in cell migration, host defense, or innate immunity [146–155]. These studies indicate that, despite its low occurrence, balancing selection has maintained functional diversity at genes involved in functions relating to the immune response, as observed for other types of selection [24, 29, 31, 77, 103].

### Tracking selection signatures from ancient DNA data

Population genetics methods can be used to estimate the approximate age and selection coefficient of adaptive mutations from data from modern human populations, with various degrees of confidence. However, the use of ancient human samples from different time periods is making it possible to determine how rapidly the frequency of adaptive mutations has increased in populations. Until a few years ago, ancient DNA data were available only for single individuals or specimens, limiting the analysis to questions of comparative genomics. We learned a great deal about the degree of admixture between modern humans and ancient hominins, such as Neanderthals and Denisovans, a topic that has been reviewed elsewhere [16, 17, 156–158]. These studies have also revealed the existence of advantageous “archaic” variants in the genomes of modern humans [16, 158]. These variants, which were acquired through admixture with archaic humans, have improved adaptation and survival in modern humans (Fig. 1c, Box 2).

However, much less is known about genetic diversity levels in populations of modern humans from different eras, such as the Paleolithic and Neolithic periods. Deep sequencing is making it possible to sequence multiple samples per species or population, opening up new possibilities for the analysis of ancient DNA data within a population genetics framework (see [156] for a review). For example, in one recent study, 230 human samples from West Eurasia dating from between 8500 and 2300 years ago were sequenced [132]. The authors searched for abrupt changes in allele frequencies over time across the genome. They identified 12 loci containing variants with frequencies that rapidly increased over time, consistent with positive selection. The lactase persistence variant yielded one of the strongest signals and appeared to have reached appreciable frequencies in Europe only recently (less than 4000 years ago), as previously suggested [131]. The other strong signals identified were either directly or indirectly related to diet, corresponding to genes encoding proteins involved in fatty acid metabolism, vitamin D levels, and celiac disease, or corresponded to genes involved in skin pigmentation [132]. Interestingly, the authors also detected strong selection signals in immunity-related genes, such as the *TLR1–TLR6–TLR10* gene cluster, which is essential for the induction of inflammatory responses and is associated with susceptibility to infectious diseases [159, 160]. Thus, ancient DNA studies can help us to understand the mode of selection following changes in human lifestyle, and the extent to which such selective events increased the frequency of functional alleles associated with specific traits or disease conditions [131, 132, 161, 162].

## Insight into rare and common diseases from natural selection

Genes associated with Mendelian or complex diseases would be expected to be subject to unequal selective pressures. We can therefore use selection signatures to predict the involvement of genes in human disease [11, 12, 32, 37, 115, 163]. Mendelian disorders are typically severe, compromising survival and reproduction, and are caused by highly penetrant, rare deleterious mutations. Mendelian disease genes should therefore fit the mutation–selection balance model, with an equilibrium between the rate of mutation and the rate of risk allele removal by purifying selection [12]. The use of population genetics models is less straightforward when it comes to predicting the genes involved in complex disease risk. Models of adaptive evolution based on positive or balancing selection apply to a few Mendelian traits or disorders, most notably, but not exclusively, those related to malaria resistance (reviewed in [76, 98]). However, the complex patterns of inheritance observed for common diseases, including incomplete penetrance, late onset and gene-by-environment interactions, make it more difficult to decipher the connection between disease risk and fitness [12].

### Purifying selection, rare variants, and severe disorders

According to population genetics theory, strongly deleterious mutations are rapidly removed from the population by purifying selection, whereas mildly deleterious mutations generally remain present, albeit at low frequencies, depending on population sizes and fitness effects. Genome-wide studies are providing increasing amounts of support for these predictions, as “essential” genes—identified as such on the basis of association with Mendelian diseases or experimental evidence from model organisms—are enriched in signs of purifying selection [32, 37, 115, 164]. Purifying selection has also been shown to be widespread in regulatory variation, acting against variants with large effects on transcription, conserved noncoding regions of the genome, and genes that are central in regulatory and protein–protein interaction networks [8, 10, 165–171].

Mutations associated with Mendelian diseases or with deleterious effects on the phenotype of the organism are generally rare and display familial segregation, but such mutations may also be restricted to specific populations [11]. This restriction, in some cases, may be due to a selective advantage provided by the disease risk allele (e.g., the sickle cell allele in populations exposed to malaria [98]), but it mostly reflects a departure from the mutation–selection balance. Small population sizes or specific demographic events may randomly increase the frequency of some disease risk alleles, because too little time has elapsed for purifying selection to remove them from the population, as observed in French Canadians, Ashkenazi Jews, or Finns [11, 66, 67].

According to these principles of population genetics, searches for genes or functional elements evolving under strong purifying selection can be used to identify the genes of major relevance for survival, mutations of which are likely to impair function and lead to severe clinical phenotypes. In this context, the immune response and host defense functions appear to be the prime targets of purifying selection [37, 95, 102]. For example, a recent study based on whole-genome sequences from the 1000 Genomes Project estimated the degree to which purifying selection acted on ~1500 innate immunity genes. The genes of this class, taken as a whole, were found to have evolved under globally stronger purifying selection than the rest of the protein-coding genome [95]. This study also assessed the strength of selective constraints in the different innate immunity modules, organizing these constraints into a hierarchy of biological relevance, and providing information about the degree to which the corresponding genes were essential or redundant [95].

Population genetics has also facilitated the identification of immune system genes and signaling pathways that fulfill essential, non-redundant functions in host defense, variants of which are associated with severe, life-threatening infectious diseases (for examples, see [94, 95, 101, 106], and for reviews [29, 103, 172, 173]). This is well illustrated by the cases of *STAT1* and *TRAF3*; they belong to the 1 % of genes presenting the strongest signals of purifying selection at the genome-wide level [95], and mutations in these genes have been associated with severe viral and bacterial diseases, Mendelian susceptibility to mycobacterial disease, and herpes simplex virus 1 encephalitis [174, 175]. Using the paradigm of immunity and infectious disease risk, these studies highlight the value of population genetics as a complement to clinical and epidemiological genetic studies, for determining the biological relevance of human genes *in natura* and in predicting their involvement in human disease [29, 103, 173, 176].

### Genetic adaptation, common variants, and complex disease

The relationship between selection and complex disease risk is less clear than for Mendelian disorders, but patterns are beginning to emerge. Genes associated with complex disease display signs of less pervasive purifying selection than Mendelian disease genes [32, 173], and are generally enriched in signals of positive selection [23, 28, 32, 37, 110, 122, 169]. There is also increasing evidence to suggest that genetic adaptations can alter complex disease susceptibility, and the population distribution of common susceptibility alleles is unlikely to result from neutral processes alone [12, 91, 177–179]. For example, the difference in susceptibility to hypertension and metabolic disorders between populations is thought to result from past adaptation to different environmental pressures [91, 179, 180]. Another study characterized the structure of complex genetic risk for 102 diseases in the context of human migration [178]. Differences between populations in the genetic risk of diseases such as type 2 diabetes, biliary liver cirrhosis, inflammatory bowel disease, systemic lupus erythematosus, and vitiligo could not be explained by simple genetic drift, providing evidence of a role for past genetic adaptation [178]. Likewise, Grossman and coworkers found overlaps between their candidate positively selected regions and genes associated with traits or diseases in GWAS [28], including height, and multiple regions associated with infectious and autoimmune disease risks, including tuberculosis and leprosy.

Like purifying selection, positive selection is prevalent among genes related to immunity and host defense [24, 37, 95, 109, 112, 115, 181]. Notable examples of immunity-related genes evolving in an adaptive manner, through different forms of positive or balancing selection, and reported to be associated with complex traits or diseases include:*TLR1* and *TLR5*, which have selection signals that seem to be related to decreases in NF-kB signaling in Europe and Africa, respectively [28, 94, 95]; many genes involved in malaria resistance in Africa and Southeast Asia [98, 100]; type-III interferon genes in Europeans and Asians, related to higher levels of spontaneous viral clearance [101, 182]; *LARGE* and *IL21*, which have been implicated in Lassa fever infectivity and immunity in West Africans [181]; and components of the NF-kB signaling pathway and inflammasome activation related to cholera resistance in a population from the Ganges river delta [97]. These cases of selection related to infectious disease and many others (see [29–31, 96, 103] for reviews and references therein) indicate that the pressures imposed by infectious disease agents have been paramount among the different threats faced by humans [183]. They also highlight the value of population genetics approaches in elucidating the variants and mechanisms underlying complex disease risk.

### Changes in selective pressures and advantageous/deleterious variants

Most of the rare and common variants associated with susceptibility to disease in modern populations have emerged through neutral selection processes [184]. However, there is increasing evidence to suggest that, following changes in environmental variables or human lifestyle, alleles that were previously adaptive can become “maladaptive” and associated with disease risk [12, 13, 29, 30, 105]. For example, according to the popular “thrifty genotype” hypothesis based on epidemiological data, the high prevalence of type 2 diabetes and obesity in modern societies results from the selection of alleles associated with efficient fat and carbohydrate storage during periods of famine in the past. Increases in food abundance and a sedentary lifestyle have rendered these alleles detrimental [185]. The strongest evidence that past selection can lead to present-day maladaptation and disease susceptibility is provided by infectious and inflammatory disorders [12, 29–31, 77, 105]. According to the hygiene hypothesis, decreases in the diversity of the microbes we are exposed to, following improvements in hygiene and the introduction of antibiotics and vaccines, have led to an imbalance in the immune response, with alleles that helped us to fight infection in the past now being associated with a higher risk of inflammation or autoimmunity [105].

Population genetics studies have provided strong support for the hygiene hypothesis, by showing that genetic variants associated with susceptibility to certain autoimmune, inflammatory, or allergic diseases, such as inflammatory bowel disease, celiac disease, type 1 diabetes, multiple sclerosis, and psoriasis, also display strong positive selection signals [29, 30, 106, 186–188]. For example, genes conferring susceptibility to inflammatory diseases have been shown to be enriched in positive selection signals, with the selected loci forming a highly interconnected protein–protein interaction network, suggesting that a shared molecular function was adaptive in the past but now affects susceptibility to various inflammatory diseases [187]. Greater protection against pathogens is thought to be the most likely driver of past selection, but it has been suggested that other traits, such as anti-inflammatory conditions *in utero*, skin color, and hypoxic responses, might account for the past selective advantage of variants, contributing to the higher frequencies of chronic disease risk alleles in current populations [30]. Additional molecular, clinical, and epidemiological studies are required to support this hypothesis, but these observations highlight, more generally, the evolutionary trade-offs between past selection and current disease risk in the context of changes in environmental pressures and human lifestyle.

## Conclusions and future directions

Population genetics offers an alternative approach, complementary to clinical and epidemiological genetic studies, for the identification of disease risk alleles/genes, the characterization of their properties, and the understanding of the relative contributions of human genetic variation to rare, severe disorders and complex disease phenotypes. Recent studies have shown that both ancient and recent demographic changes have modified the burden of rare, deleterious variants segregating in the population, whereas the population frequencies of other variants have increased because they conferred advantages in terms of better survival and reproduction.

These studies have made a major contribution, but further theoretical and empirical work is needed. Rare-variant studies should consider different fitness and dominance effects, epistatic interactions, and detailed demographic modeling to evaluate the potential impact of local changes in population size and admixture on the efficiency of purifying selection. Furthermore, rare-variant association studies involving complex traits or diseases should seek to account for the evolutionary forces that affect genetic architecture, such as selection and population demography, and integrate elaborated models of population genetics that consider the relationship between allele frequency and effect size and the distribution of phenotypes, as recently reported [189]. Independently of the complex interactions between demography and selection, additional sequence-based studies are required to catalog rare variants in different worldwide populations (including isolated populations), focusing not only on point mutations but also on indels, inversions, or copy-number variation, and evaluate their contribution to disease risk.

Studies of genetic adaptation, particularly those aiming to make connections with disease in populations historically exposed to different environmental variables, should generate whole-genome data for different worldwide populations with greatly contrasting demographic histories, lifestyles, and subsistence strategies. There is also a need to develop and improve statistical approaches to facilitate the detection of positive selection following alternative modes of genetic adaptation, such as selection on standing variation, polygenic adaptation, and adaptive introgression. These selection studies, if combined with data for molecular phenotypes (e.g., gene expression, protein and metabolite levels, epigenetic marks) and organismal phenotypes (in health and disease), should provide great insight into adaptive phenotypes of major relevance in human evolution and the genetic architecture of rare and common human diseases.

## Box 1. To what extent has positive selection continued in recent times?

Many human traits related to healthy or disease conditions appear to have conferred selective advantages in the past [23–28, 71, 109], but the extent to which selection has persisted in very recent times remains largely unknown. This is because current methods of detecting positive selection are designed to measure selection over long evolutionary timescales [24, 26]. A recent study has developed a new method, the Singleton Density Score (SDS), to infer recent changes in allele frequencies using modern whole-genome sequence data, and to measure selection during the past 2000 years [190]. Using simulations, they first showed that SDS has equal power to detect hard and soft sweeps within recent evolutionary timescales. By applying SDS to more than 3000 full genomes of British ancestry, they identified several alleles and traits that have provided, or continue to provide, a strong selective advantage over the last 2000 years. Among the strongest hits, they found the lactase persistence allele, indicating that the known selective advantage provided by this allele [78, 81] has persisted in very recent times. They also detected strong signals of recent positive selection in the HLA region (associated with multiple traits and diseases), in genes involved in skin pigmentation (likely favoring blond hair and blue eyes through sexual selection), and some cases of recent polygenic adaptation favoring increased height, increased infant head circumference and birth weight, later sexual maturation in women, and decreased levels of insulin and glycated hemoglobin [190]. This study provides a new method to test the extent to which selection on complex traits, either following the hard-sweep model or reflecting polygenic adaptation, has shaped phenotypic diversity in other human populations within historical times.

## Box 2. Archaic admixture and disease risk in modern humans

One source of adaptive genetic variation is variants from donor populations that have undergone introgression into recipient populations (or species) through interbreeding or admixture, a phenomenon known as adaptive introgression. During the dispersals of modern humans through Eurasia, they encountered other human forms, such as Neanderthals and Denisovans [16, 17, 156–158]. Analyses of whole-genome sequences from these hominins [191, 192] have shown that admixture between modern and archaic humans occurred, with modern Eurasian genomes comprising 1–6 % of Neanderthal or Denisovan ancestry (Fig. 1c) [191, 193–197]. However, these estimations are averaged across the genome, and it has been shown that the degree of archaic ancestry in the modern human genome varies considerably [194, 198, 199]. A systematic search for regions of Neanderthal ancestry in the genomes of modern humans has shown that, globally, purifying selection has acted against Neanderthal introgression, particularly in protein-coding regions [194]. However, some regions of the genome can present a high degree of Neanderthal ancestry, which can be as high as 62 % in Asian and 64 % in European populations [194].

Some of the regions presenting the highest degree of Neanderthal ancestry have been found to overlap alleles that have been associated with phenotypes of medical relevance, such as lupus, biliary cirrhosis, Crohn’s disease, optic-disk size, smoking behavior, IL-18 levels, type 2 diabetes, and hypercoagulation [194, 200]. In some cases, the introgression of archaic segments into the genomes of modern humans appears to have been adaptive [194, 198]. Cases of adaptive introgression from Neanderthals or Denisovans have been reported for several genes (see [16] for a complete list), such as *EPAS1*, involved in human adaptation to life at high altitude [201], *BNC2*, involved in skin pigmentation [194, 198], and an increasing number of genes involved in immunity to infection, including several *HLA* genes [202], *STAT2* [203], the *OAS* gene cluster [204, 205], and the *TLR6*-*TLR1*-*TLR10* gene cluster [95, 206]. Collectively, these studies suggest that archaic admixture has been an important source of adaptive variation but also that modern humans have inherited archaic variation that today influences complex disease risk [158, 200].

## Abbreviations

*BNC2*: Basonuclin-2
*DARC*: Duffy antigen/chemokine receptor
eQTL: expression quantitative trait loci
ExAC: Exome Aggregation Consortium
*FUT2*: Fucosyltransferase 2
*G6PD*: Glucose-6-phosphate dehydrogenase
GWAS: Genome-wide association studies
HbS: sickle cell haemoglobin polymorphism
HLA: Human leukocyte antigen
*IGFBP7*: Insulin-like growth factor-binding protein 7
*IL21*: Interleukin 21
*KIR*: Killer-cell immunoglobulin-like receptors
*LARGE*: LARGE xylosyl- and glucuronyltransferase 1
MHC: Major histocompatibility complex
NF-kB: nuclear factor NF-κB
*OAS*: O-Acetylserine
SDS: singleton density score
*STAT1*: Signal transducer and activator of transcription 1
*TLR*: Toll-like receptors
*TRAF3*: Tumor necrosis factor receptor-associated factor

## References

[1] Pritchard JK (2001). Are rare variants responsible for susceptibility to complex diseases?. Am J Hum Genet.

[2] Pritchard JK, Cox NJ (2002). The allelic architecture of human disease genes: common disease-common variant…or not?. Hum Mol Genet.

[3] Manolio TA, Collins FS, Cox NJ, Goldstein DB, Hindorff LA, Hunter DJ (2009). Finding the missing heritability of complex diseases. Nature.

[4] McCarthy MI, Abecasis GR, Cardon LR, Goldstein DB, Little J, Ioannidis JP (2008). Genome-wide association studies for complex traits: consensus, uncertainty and challenges. Nat Rev Genet.

[5] Reich DE, Lander ES (2001). On the allelic spectrum of human disease. Trends Genet.

[6] Zwick ME, Cutler DJ, Chakravarti A (2000). Patterns of genetic variation in Mendelian and complex traits. Annu Rev Genomics Hum Genet.

[7] Schork NJ, Murray SS, Frazer KA, Topol EJ (2009). Common vs. rare allele hypotheses for complex diseases. Curr Opin Genet Dev.

[8] Bodmer W, Bonilla C (2008). Common and rare variants in multifactorial susceptibility to common diseases. Nat Genet.

[9] Goldstein DB (2009). Common genetic variation and human traits. N Engl J Med.

[10] Zhu Q, Ge D, Maia JM, Zhu M, Petrovski S, Dickson SP (2011). A genome-wide comparison of the functional properties of rare and common genetic variants in humans. Am J Hum Genet.

[11] Lu YF, Goldstein DB, Angrist M, Cavalleri G (2014). Personalized medicine and human genetic diversity. Cold Spring Harb Perspect Med.

[12] Di Rienzo A (2006). Population genetics models of common diseases. Curr Opin Genet Dev.

[13] Crespi BJ (2011). The emergence of human-evolutionary medical genomics. Evol Appl.

[14] Abecasis GR, Auton A, Brooks LD, DePristo MA, Durbin RM, Handsaker RE (2012). An integrated map of genetic variation from 1,092 human genomes. Nature.

[15] 1000 Genomes Project Consortium (2015). A global reference for human genetic variation. Nature.

[16] Racimo F, Sankararaman S, Nielsen R, Huerta-Sanchez E (2015). Evidence for archaic adaptive introgression in humans. Nat Rev Genet.

[17] Kelso J, Prufer K (2014). Ancient humans and the origin of modern humans. Curr Opin Genet Dev.

[18] Veeramah KR, Hammer MF (2014). The impact of whole-genome sequencing on the reconstruction of human population history. Nat Rev Genet.

[19] Novembre J, Ramachandran S (2011). Perspectives on human population structure at the cusp of the sequencing era. Annu Rev Genomics Hum Genet.

[20] Henn BM, Cavalli-Sforza LL, Feldman MW (2012). The great human expansion. Proc Natl Acad Sci U S A.

[21] Sousa V, Peischl S, Excoffier L (2014). Impact of range expansions on current human genomic diversity. Curr Opin Genet Dev.

[22] Lohmueller KE (2014). The distribution of deleterious genetic variation in human populations. Curr Opin Genet Dev.

[23] Nielsen R, Hellmann I, Hubisz M, Bustamante C, Clark AG (2007). Recent and ongoing selection in the human genome. Nat Rev Genet.

[24] Sabeti PC, Schaffner SF, Fry B, Lohmueller J, Varilly P, Shamovsky O (2006). Positive natural selection in the human lineage. Science.

[25] Jeong C, Di Rienzo A (2014). Adaptations to local environments in modern human populations. Curr Opin Genet Dev.

[26] Vitti JJ, Grossman SR, Sabeti PC (2013). Detecting natural selection in genomic data. Annu Rev Genet.

[27] Key FM, Teixeira JC, de Filippo C, Andres AM (2014). Advantageous diversity maintained by balancing selection in humans. Curr Opin Genet Dev.

[28] Grossman SR, Andersen KG, Shlyakhter I, Tabrizi S, Winnicki S, Yen A (2013). Identifying recent adaptations in large-scale genomic data. Cell.

[29] Barreiro LB, Quintana-Murci L (2010). From evolutionary genetics to human immunology: how selection shapes host defence genes. Nat Rev Genet.

[30] Brinkworth JF, Barreiro LB (2014). The contribution of natural selection to present-day susceptibility to chronic inflammatory and autoimmune disease. Curr Opin Immunol.

[31] Karlsson EK, Kwiatkowski DP, Sabeti PC (2014). Natural selection and infectious disease in human populations. Nat Rev Genet.

[32] Blekhman R, Man O, Herrmann L, Boyko AR, Indap A, Kosiol C (2008). Natural selection on genes that underlie human disease susceptibility. Curr Biol.

[33] Eyre-Walker A, Keightley PD (1999). High genomic deleterious mutation rates in hominids. Nature.

[34] Kryukov GV, Pennacchio LA, Sunyaev SR (2007). Most rare missense alleles are deleterious in humans: implications for complex disease and association studies. Am J Hum Genet.

[35] Boyko AR, Williamson SH, Indap AR, Degenhardt JD, Hernandez RD, Lohmueller KE (2008). Assessing the evolutionary impact of amino acid mutations in the human genome. PLoS Genet.

[36] Eyre-Walker A, Keightley PD (2007). The distribution of fitness effects of new mutations. Nat Rev Genet.

[37] Bustamante CD, Fledel-Alon A, Williamson S, Nielsen R, Hubisz MT, Glanowski S (2005). Natural selection on protein-coding genes in the human genome. Nature.

[38] Kimura M, Maruyama T, Crow JF (1963). The mutation load in small populations. Genetics.

[39] Ohta T (1973). Slightly deleterious mutant substitutions in evolution. Nature.

[40] Akashi H, Osada N, Ohta T (2012). Weak selection and protein evolution. Genetics.

[41] Coventry A, Bull-Otterson LM, Liu X, Clark AG, Maxwell TJ, Crosby J (2010). Deep resequencing reveals excess rare recent variants consistent with explosive population growth. Nat Commun.

[42] Marth GT, Yu F, Indap AR, Garimella K, Gravel S, Leong WF (2011). The functional spectrum of low-frequency coding variation. Genome Biol.

[43] Keinan A, Clark AG (2012). Recent explosive human population growth has resulted in an excess of rare genetic variants. Science.

[44] Nelson MR, Wegmann D, Ehm MG, Kessner D, St Jean P, Verzilli C (2012). An abundance of rare functional variants in 202 drug target genes sequenced in 14,002 people. Science.

[45] Tennessen JA, Bigham AW, O’Connor TD, Fu W, Kenny EE, Gravel S (2012). Evolution and functional impact of rare coding variation from deep sequencing of human exomes. Science.

[46] Fu W, O’Connor TD, Jun G, Kang HM, Abecasis G, Leal SM (2013). Analysis of 6,515 exomes reveals the recent origin of most human protein-coding variants. Nature.

[47] Lek M, Karczewski KJ, Minikel EV, Samocha KE, Banks E, Fennell T (2016). Analysis of protein-coding genetic variation in 60,706 humans. Nature.

[48] Agarwala V, Flannick J, Sunyaev S, Go TDC, Altshuler D (2013). Evaluating empirical bounds on complex disease genetic architecture. Nat Genet.

[49] Gibson G (2011). Rare and common variants: twenty arguments. Nat Rev Genet.

[50] Maher MC, Uricchio LH, Torgerson DG, Hernandez RD (2012). Population genetics of rare variants and complex diseases. Hum Hered.

[51] Gravel S, Henn BM, Gutenkunst RN, Indap AR, Marth GT, Clark AG (2011). Demographic history and rare allele sharing among human populations. Proc Natl Acad Sci U S A.

[52] Lohmueller KE, Indap AR, Schmidt S, Boyko AR, Hernandez RD, Hubisz MJ (2008). Proportionally more deleterious genetic variation in European than in African populations. Nature.

[53] Peischl S, Dupanloup I, Kirkpatrick M, Excoffier L (2013). On the accumulation of deleterious mutations during range expansions. Mol Ecol.

[54] Eyre-Walker A (2010). Evolution in health and medicine Sackler colloquium: genetic architecture of a complex trait and its implications for fitness and genome-wide association studies. Proc Natl Acad Sci U S A.

[55] Adzhubei IA, Schmidt S, Peshkin L, Ramensky VE, Gerasimova A, Bork P (2010). A method and server for predicting damaging missense mutations. Nat Methods.

[56] Cooper GM, Stone EA, Asimenos G, Program NCS, Green ED, Batzoglou S (2005). Distribution and intensity of constraint in mammalian genomic sequence. Genome Res.

[57] Kumar P, Henikoff S, Ng PC (2009). Predicting the effects of coding non-synonymous variants on protein function using the SIFT algorithm. Nat Protoc.

[58] Dong C, Wei P, Jian X, Gibbs R, Boerwinkle E, Wang K (2015). Comparison and integration of deleteriousness prediction methods for nonsynonymous SNVs in whole exome sequencing studies. Hum Mol Genet.

[59] Kircher M, Witten DM, Jain P, O’Roak BJ, Cooper GM, Shendure J (2014). A general framework for estimating the relative pathogenicity of human genetic variants. Nat Genet.

[60] Itan Y, Shang L, Boisson B, Ciancanelli MJ, Markle JG, Martinez-Barricarte R (2016). The mutation significance cutoff: gene-level thresholds for variant predictions. Nat Methods.

[61] Gutenkunst RN, Hernandez RD, Williamson SH, Bustamante CD (2009). Inferring the joint demographic history of multiple populations from multidimensional SNP frequency data. PLoS Genet.

[62] Do R, Balick D, Li H, Adzhubei I, Sunyaev S, Reich D (2015). No evidence that selection has been less effective at removing deleterious mutations in Europeans than in Africans. Nat Genet.

[63] Fu W, Gittelman RM, Bamshad MJ, Akey JM (2014). Characteristics of neutral and deleterious protein-coding variation among individuals and populations. Am J Hum Genet.

[64] Simons YB, Turchin MC, Pritchard JK, Sella G (2014). The deleterious mutation load is insensitive to recent population history. Nat Genet.

[65] Henn BM, Botigue LR, Bustamante CD, Clark AG, Gravel S (2015). Estimating the mutation load in human genomes. Nat Rev Genet.

[66] Casals F, Hodgkinson A, Hussin J, Idaghdour Y, Bruat V, de Maillard T (2013). Whole-exome sequencing reveals a rapid change in the frequency of rare functional variants in a founding population of humans. PLoS Genet.

[67] Lim ET, Wurtz P, Havulinna AS, Palta P, Tukiainen T, Rehnstrom K (2014). Distribution and medical impact of loss-of-function variants in the Finnish founder population. PLoS Genet.

[68] Henn BM, Botigue LR, Peischl S, Dupanloup I, Lipatov M, Maples BK (2016). Distance from sub-Saharan Africa predicts mutational load in diverse human genomes. Proc Natl Acad Sci U S A.

[69] Klopfstein S, Currat M, Excoffier L (2006). The fate of mutations surfing on the wave of a range expansion. Mol Biol Evol.

[70] Lohmueller KE (2014). The impact of population demography and selection on the genetic architecture of complex traits. PLoS Genet.

[71] Segurel L, Quintana-Murci L (2014). Preserving immune diversity through ancient inheritance and admixture. Curr Opin Immunol.

[72] Scheinfeldt LB, Tishkoff SA (2013). Recent human adaptation: genomic approaches, interpretation and insights. Nat Rev Genet.

[73] Pritchard JK, Di Rienzo A (2010). Adaptation—not by sweeps alone. Nat Rev Genet.

[74] Pritchard JK, Pickrell JK, Coop G (2010). The genetics of human adaptation: hard sweeps, soft sweeps, and polygenic adaptation. Curr Biol.

[75] Harris EE, Meyer D. The molecular signature of selection underlying human adaptations. Am J Phys Anthropol. 2006;Suppl 43:89–130 10.1002/ajpa.20518 17103426

[76] Quintana-Murci L, Barreiro LB (2010). The role played by natural selection on Mendelian traits in humans. Ann N Y Acad Sci.

[77] Siddle KJ, Quintana-Murci L (2014). The Red Queen’s long race: human adaptation to pathogen pressure. Curr Opin Genet Dev.

[78] Bersaglieri T, Sabeti PC, Patterson N, Vanderploeg T, Schaffner SF, Drake JA (2004). Genetic signatures of strong recent positive selection at the lactase gene. Am J Hum Genet.

[79] Tishkoff SA, Reed FA, Ranciaro A, Voight BF, Babbitt CC, Silverman JS (2007). Convergent adaptation of human lactase persistence in Africa and Europe. Nat Genet.

[80] Enattah NS, Jensen TG, Nielsen M, Lewinski R, Kuokkanen M, Rasinpera H (2008). Independent introduction of two lactase-persistence alleles into human populations reflects different history of adaptation to milk culture. Am J Hum Genet.

[81] Itan Y, Powell A, Beaumont MA, Burger J, Thomas MG (2009). The origins of lactase persistence in Europe. PLoS Comput Biol.

[82] Ranciaro A, Campbell MC, Hirbo JB, Ko WY, Froment A, Anagnostou P (2014). Genetic origins of lactase persistence and the spread of pastoralism in Africa. Am J Hum Genet.

[83] Beleza S, Santos AM, McEvoy B, Alves I, Martinho C, Cameron E (2013). The timing of pigmentation lightening in Europeans. Mol Biol Evol.

[84] Miller CT, Beleza S, Pollen AA, Schluter D, Kittles RA, Shriver MD (2007). cis-Regulatory changes in Kit ligand expression and parallel evolution of pigmentation in sticklebacks and humans. Cell.

[85] Norton HL, Kittles RA, Parra E, McKeigue P, Mao X, Cheng K (2007). Genetic evidence for the convergent evolution of light skin in Europeans and East Asians. Mol Biol Evol.

[86] Lamason RL, Mohideen MA, Mest JR, Wong AC, Norton HL, Aros MC (2005). SLC24A5, a putative cation exchanger, affects pigmentation in zebrafish and humans. Science.

[87] Hancock AM, Witonsky DB, Alkorta-Aranburu G, Beall CM, Gebremedhin A, Sukernik R (2011). Adaptations to climate-mediated selective pressures in humans. PLoS Genet.

[88] Yi X, Liang Y, Huerta-Sanchez E, Jin X, Cuo ZX, Pool JE (2010). Sequencing of 50 human exomes reveals adaptation to high altitude. Science.

[89] Bigham A, Bauchet M, Pinto D, Mao X, Akey JM, Mei R (2010). Identifying signatures of natural selection in Tibetan and Andean populations using dense genome scan data. PLoS Genet.

[90] Simonson TS, Yang Y, Huff CD, Yun H, Qin G, Witherspoon DJ (2010). Genetic evidence for high-altitude adaptation in Tibet. Science.

[91] Hancock AM, Witonsky DB, Gordon AS, Eshel G, Pritchard JK, Coop G (2008). Adaptations to climate in candidate genes for common metabolic disorders. PLoS Genet.

[92] Alkorta-Aranburu G, Beall CM, Witonsky DB, Gebremedhin A, Pritchard JK, Di Rienzo A (2012). The genetic architecture of adaptations to high altitude in Ethiopia. PLoS Genet.

[93] Coop G, Pickrell JK, Novembre J, Kudaravalli S, Li J, Absher D (2009). The role of geography in human adaptation. PLoS Genet.

[94] Barreiro LB, Ben-Ali M, Quach H, Laval G, Patin E, Pickrell JK (2009). Evolutionary dynamics of human Toll-like receptors and their different contributions to host defense. PLoS Genet.

[95] Deschamps M, Laval G, Fagny M, Itan Y, Abel L, Casanova JL (2016). Genomic signatures of selective pressures and introgression from archaic hominins at human innate immunity genes. Am J Hum Genet.

[96] Fumagalli M, Sironi M (2014). Human genome variability, natural selection and infectious diseases. Curr Opin Immunol.

[97] Karlsson EK, Harris JB, Tabrizi S, Rahman A, Shlyakhter I, Patterson N (2013). Natural selection in a bangladeshi population from the cholera-endemic ganges river delta. Sci Transl Med.

[98] Kwiatkowski DP (2005). How malaria has affected the human genome and what human genetics can teach us about malaria. Am J Hum Genet.

[99] Laayouni H, Oosting M, Luisi P, Ioana M, Alonso S, Ricano-Ponce I (2014). Convergent evolution in European and Rroma populations reveals pressure exerted by plague on Toll-like receptors. Proc Natl Acad Sci U S A.

[100] Louicharoen C, Patin E, Paul R, Nuchprayoon I, Witoonpanich B, Peerapittayamongkol C (2009). Positively selected G6PD-Mahidol mutation reduces Plasmodium vivax density in Southeast Asians. Science.

[101] Manry J, Laval G, Patin E, Fornarino S, Itan Y, Fumagalli M (2011). Evolutionary genetic dissection of human interferons. J Exp Med.

[102] Mukherjee S, Sarkar-Roy N, Wagener DK, Majumder PP (2009). Signatures of natural selection are not uniform across genes of innate immune system, but purifying selection is the dominant signature. Proc Natl Acad Sci U S A.

[103] Quintana-Murci L, Clark AG (2013). Population genetic tools for dissecting innate immunity in humans. Nat Rev Immunol.

[104] Sabeti PC, Reich DE, Higgins JM, Levine HZ, Richter DJ, Schaffner SF (2002). Detecting recent positive selection in the human genome from haplotype structure. Nature.

[105] Sironi M, Clerici M (2010). The hygiene hypothesis: an evolutionary perspective. Microbes Infect.

[106] Vasseur E, Boniotto M, Patin E, Laval G, Quach H, Manry J (2012). The evolutionary landscape of cytosolic microbial sensors in humans. Am J Hum Genet.

[107] Wlasiuk G, Nachman MW (2010). Adaptation and constraint at Toll-like receptors in primates. Mol Biol Evol.

[108] Jeong C, Alkorta-Aranburu G, Basnyat B, Neupane M, Witonsky DB, Pritchard JK (2014). Admixture facilitates genetic adaptations to high altitude in Tibet. Nat Communs.

[109] Pickrell JK, Coop G, Novembre J, Kudaravalli S, Li JZ, Absher D (2009). Signals of recent positive selection in a worldwide sample of human populations. Genome Res.

[110] Sabeti PC, Varilly P, Fry B, Lohmueller J, Hostetter E, Cotsapas C (2007). Genome-wide detection and characterization of positive selection in human populations. Nature.

[111] Tang K, Thornton KR, Stoneking M (2007). A new approach for using genome scans to detect recent positive selection in the human genome. PLoS Biol.

[112] Voight BF, Kudaravalli S, Wen X, Pritchard JK (2006). A map of recent positive selection in the human genome. PLoS Biol.

[113] Carlson CS, Thomas DJ, Eberle MA, Swanson JE, Livingston RJ, Rieder MJ (2005). Genomic regions exhibiting positive selection identified from dense genotype data. Genome Res.

[114] Kelley JL, Madeoy J, Calhoun JC, Swanson W, Akey JM (2006). Genomic signatures of positive selection in humans and the limits of outlier approaches. Genome Res.

[115] Barreiro LB, Laval G, Quach H, Patin E, Quintana-Murci L (2008). Natural selection has driven population differentiation in modern humans. Nat Genet.

[116] Chen H, Patterson N, Reich D (2010). Population differentiation as a test for selective sweeps. Genome Res.

[117] Jin W, Xu S, Wang H, Yu Y, Shen Y, Wu B (2012). Genome-wide detection of natural selection in African Americans pre- and post-admixture. Genome Res.

[118] Weir BS, Cardon LR, Anderson AD, Nielsen DM, Hill WG (2005). Measures of human population structure show heterogeneity among genomic regions. Genome Res.

[119] Akey JM, Zhang G, Zhang K, Jin L, Shriver MD (2002). Interrogating a high-density SNP map for signatures of natural selection. Genome Res.

[120] Akey JM (2009). Constructing genomic maps of positive selection in humans: where do we go from here?. Genome Res.

[121] Williamson SH, Hubisz MJ, Clark AG, Payseur BA, Bustamante CD, Nielsen R (2007). Localizing recent adaptive evolution in the human genome. PLoS Genet.

[122] Fagny M, Patin E, Enard D, Barreiro LB, Quintana-Murci L, Laval G (2014). Exploring the occurrence of classic selective sweeps in humans using whole-genome sequencing data sets. Mol Biol Evol.

[123] Hernandez RD, Kelley JL, Elyashiv E, Melton SC, Auton A, McVean G (2011). Classic selective sweeps were rare in recent human evolution. Science.

[124] Granka JM, Henn BM, Gignoux CR, Kidd JM, Bustamante CD, Feldman MW (2012). Limited evidence for classic selective sweeps in African populations. Genetics.

[125] Vernot B, Stergachis AB, Maurano MT, Vierstra J, Neph S, Thurman RE (2012). Personal and population genomics of human regulatory variation. Genome Res.

[126] Fraser HB (2013). Gene expression drives local adaptation in humans. Genome Res.

[127] Pickrell JK (2014). Joint analysis of functional genomic data and genome-wide association studies of 18 human traits. Am J Hum Genet.

[128] Schaub MA, Boyle AP, Kundaje A, Batzoglou S, Snyder M (2012). Linking disease associations with regulatory information in the human genome. Genome Res.

[129] Nakagome S, Alkorta-Aranburu G, Amato R, Howie B, Peter BM, Hudson RR (2016). Estimating the ages of selection signals from different epochs in human history. Mol Biol Evol.

[130] Peter BM, Huerta-Sanchez E, Nielsen R (2012). Distinguishing between selective sweeps from standing variation and from a de novo mutation. PLoS Genet.

[131] Allentoft ME, Sikora M, Sjogren KG, Rasmussen S, Rasmussen M, Stenderup J (2015). Population genomics of Bronze Age Eurasia. Nature.

[132] Mathieson I, Lazaridis I, Rohland N, Mallick S, Patterson N, Roodenberg SA (2015). Genome-wide patterns of selection in 230 ancient Eurasians. Nature.

[133] Berg JJ, Coop G (2014). A population genetic signal of polygenic adaptation. PLoS Genet.

[134] Turchin MC, Chiang CW, Palmer CD, Sankararaman S, Reich D, Hirschhorn JN (2012). Evidence of widespread selection on standing variation in Europe at height-associated SNPs. Nat Genet.

[135] Messer PW, Petrov DA (2013). Population genomics of rapid adaptation by soft selective sweeps. Trends Ecol Evol.

[136] Charlesworth D (2006). Balancing selection and its effects on sequences in nearby genome regions. PLoS Genet.

[137] Klein J, Sato A, Nagl S, O’HUigin C (1998). Molecular trans-species polymorphism. Annu Rev Ecol Syst.

[138] Allison AC (1954). Protection afforded by sickle-cell trait against subtertian malareal infection. Br Med J.

[139] Klein J, Satta Y, O’HUigin C, Takahata N (1993). The molecular descent of the major histocompatibility complex. Annu Rev Immunol.

[140] Hughes AL, Nei M (1988). Pattern of nucleotide substitution at major histocompatibility complex class I loci reveals overdominant selection. Nature.

[141] Prugnolle F, Manica A, Charpentier M, Guegan JF, Guernier V, Balloux F (2005). Pathogen-driven selection and worldwide HLA class I diversity. Curr Biol.

[142] Segurel L, Thompson EE, Flutre T, Lovstad J, Venkat A, Margulis SW (2012). The ABO blood group is a trans-species polymorphism in primates. Proc Natl Acad Sci U S A.

[143] Cagliani R, Guerini FR, Fumagalli M, Riva S, Agliardi C, Galimberti D (2012). A trans-specific polymorphism in ZC3HAV1 is maintained by long-standing balancing selection and may confer susceptibility to multiple sclerosis. Mol Biol Evol.

[144] Leffler EM, Gao Z, Pfeifer S, Segurel L, Auton A, Venn O (2013). Multiple instances of ancient balancing selection shared between humans and chimpanzees. Science.

[145] Teixeira JC, de Filippo C, Weihmann A, Meneu JR, Racimo F, Dannemann M (2015). Long-term balancing selection in LAD1 maintains a missense trans-species polymorphism in humans, chimpanzees, and bonobos. Mol Biol Evol.

[146] Single RM, Martin MP, Gao X, Meyer D, Yeager M, Kidd JR (2007). Global diversity and evidence for coevolution of KIR and HLA. Nat Genet.

[147] Andres AM, Hubisz MJ, Indap A, Torgerson DG, Degenhardt JD, Boyko AR (2009). Targets of balancing selection in the human genome. Mol Biol Evol.

[148] DeGiorgio M, Lohmueller KE, Nielsen R (2014). A model-based approach for identifying signatures of ancient balancing selection in genetic data. PLoS Genet.

[149] Rasmussen MD, Hubisz MJ, Gronau I, Siepel A (2014). Genome-wide inference of ancestral recombination graphs. PLoS Genet.

[150] Ferrer-Admetlla A, Bosch E, Sikora M, Marques-Bonet T, Ramirez-Soriano A, Muntasell A (2008). Balancing selection is the main force shaping the evolution of innate immunity genes. J Immunol.

[151] Bronson PG, Mack SJ, Erlich HA, Slatkin M (2013). A sequence-based approach demonstrates that balancing selection in classical human leukocyte antigen (HLA) loci is asymmetric. Hum Mol Genet.

[152] Andres AM, Dennis MY, Kretzschmar WW, Cannons JL, Lee-Lin SQ, Hurle B (2010). Balancing selection maintains a form of ERAP2 that undergoes nonsense-mediated decay and affects antigen presentation. PLoS Genet.

[153] Norman PJ, Abi-Rached L, Gendzekhadze K, Korbel D, Gleimer M, Rowley D (2007). Unusual selection on the KIR3DL1/S1 natural killer cell receptor in Africans. Nat Genet.

[154] Fumagalli M, Fracassetti M, Cagliani R, Forni D, Pozzoli U, Comi GP (2012). An evolutionary history of the selectin gene cluster in humans. Heredity (Edinb).

[155] Hollox EJ, Armour JA (2008). Directional and balancing selection in human beta-defensins. BMC Evol Biol.

[156] Leonardi M, Librado P, Der Sarkissian C, Schubert M, Alfarhan AH, Alquraishi SA, et al. Evolutionary patterns and processes: lessons from ancient DNA. Syst Biol. 2016. doi: 10.1093/sysbio/syw059 10.1093/sysbio/syw059 PMC5410953 28173586

[157] Haber M, Mezzavilla M, Xue Y, Tyler-Smith C (2016). Ancient DNA and the rewriting of human history: be sparing with Occam’s razor. Genome Biol.

[158] Vattathil S, Akey JM (2015). Small amounts of archaic admixture provide big insights into human history. Cell.

[159] Wong SH, Gochhait S, Malhotra D, Pettersson FH, Teo YY, Khor CC (2010). Leprosy and the adaptation of human toll-like receptor 1. PLoS Pathog.

[160] Uciechowski P, Imhoff H, Lange C, Meyer CG, Browne EN, Kirsten DK (2011). Susceptibility to tuberculosis is associated with TLR1 polymorphisms resulting in a lack of TLR1 cell surface expression. J Leukoc Biol.

[161] Broushaki F, Thomas MG, Link V, Lopez S, van Dorp L, Kirsanow K (2016). Early Neolithic genomes from the eastern Fertile Crescent. Science.

[162] Hofmanova Z, Kreutzer S, Hellenthal G, Sell C, Diekmann Y, Diez-Del-Molino D (2016). Early farmers from across Europe directly descended from Neolithic Aegeans. Proc Natl Acad Sci U S A.

[163] Nielsen R, Hubisz MJ, Hellmann I, Torgerson D, Andres AM, Albrechtsen A (2009). Darwinian and demographic forces affecting human protein coding genes. Genome Res.

[164] Georgi B, Voight BF, Bucan M (2013). From mouse to human: evolutionary genomics analysis of human orthologs of essential genes. PLoS Genet.

[165] Battle A, Mostafavi S, Zhu X, Potash JB, Weissman MM, McCormick C (2014). Characterizing the genetic basis of transcriptome diversity through RNA-sequencing of 922 individuals. Genome Res.

[166] Gerstein MB, Kundaje A, Hariharan M, Landt SG, Yan KK, Cheng C (2012). Architecture of the human regulatory network derived from ENCODE data. Nature.

[167] Fraser HB, Hirsh AE, Steinmetz LM, Scharfe C, Feldman MW (2002). Evolutionary rate in the protein interaction network. Science.

[168] Jordan IK, Marino-Ramirez L, Wolf YI, Koonin EV (2004). Conservation and coevolution in the scale-free human gene coexpression network. Mol Biol Evol.

[169] Torgerson DG, Boyko AR, Hernandez RD, Indap A, Hu X, White TJ (2009). Evolutionary processes acting on candidate cis-regulatory regions in humans inferred from patterns of polymorphism and divergence. PLoS Genet.

[170] Katzman S, Kern AD, Bejerano G, Fewell G, Fulton L, Wilson RK (2007). Human genome ultraconserved elements are ultraselected. Science.

[171] Drake JA, Bird C, Nemesh J, Thomas DJ, Newton-Cheh C, Reymond A (2006). Conserved noncoding sequences are selectively constrained and not mutation cold spots. Nat Genet.

[172] Casanova JL, Abel L, Quintana-Murci L (2011). Human TLRs and IL-1Rs in host defense: natural insights from evolutionary, epidemiological, and clinical genetics. Annu Rev Immunol.

[173] Alcais A, Quintana-Murci L, Thaler DS, Schurr E, Abel L, Casanova JL (2010). Life-threatening infectious diseases of childhood: single-gene inborn errors of immunity?. Ann N Y Acad Sci.

[174] Boisson-Dupuis S, Kong XF, Okada S, Cypowyj S, Puel A, Abel L (2012). Inborn errors of human STAT1: allelic heterogeneity governs the diversity of immunological and infectious phenotypes. Curr Opin Immunol.

[175] Perez de Diego R, Sancho-Shimizu V, Lorenzo L, Puel A, Plancoulaine S, Picard C (2010). Human TRAF3 adaptor molecule deficiency leads to impaired Toll-like receptor 3 response and susceptibility to herpes simplex encephalitis. Immunity.

[176] Casanova JL, Abel L, Quintana-Murci L (2013). Immunology taught by human genetics. Cold Spring Harb Symp Quant Biol.

[177] Colonna V, Ayub Q, Chen Y, Pagani L, Luisi P, Pybus M (2014). Human genomic regions with exceptionally high levels of population differentiation identified from 911 whole-genome sequences. Genome Biol.

[178] Corona E, Chen R, Sikora M, Morgan AA, Patel CJ, Ramesh A (2013). Analysis of the genetic basis of disease in the context of worldwide human relationships and migration. PLoS Genet.

[179] Young JH, Chang YP, Kim JD, Chretien JP, Klag MJ, Levine MA (2005). Differential susceptibility to hypertension is due to selection during the out-of-Africa expansion. PLoS Genet.

[180] Chen R, Corona E, Sikora M, Dudley JT, Morgan AA, Moreno-Estrada A (2012). Type 2 diabetes risk alleles demonstrate extreme directional differentiation among human populations, compared to other diseases. PLoS Genet.

[181] Andersen KG, Shylakhter I, Tabrizi S, Grossman SR, Happi CT, Sabeti PC (2012). Genome-wide scans provide evidence for positive selection of genes implicated in Lassa fever. Philos Trans R Soc Lond B Biol Sci.

[182] Key FM, Peter B, Dennis MY, Huerta-Sanchez E, Tang W, Prokunina-Olsson L (2014). Selection on a variant associated with improved viral clearance drives local, adaptive pseudogenization of interferon lambda 4 (IFNL4). PLoS Genet.

[183] Fumagalli M, Sironi M, Pozzoli U, Ferrer-Admetlla A, Pattini L, Nielsen R (2011). Signatures of environmental genetic adaptation pinpoint pathogens as the main selective pressure through human evolution. PLoS Genet.

[184] Dudley JT, Kim Y, Liu L, Markov GJ, Gerold K, Chen R (2012). Human genomic disease variants: a neutral evolutionary explanation. Genome Res.

[185] Neel JV (1962). Diabetes mellitus: a “thrifty” genotype rendered detrimental by “progress”?. Am J Hum Genet.

[186] Fumagalli M, Pozzoli U, Cagliani R, Comi GP, Riva S, Clerici M (2009). Parasites represent a major selective force for interleukin genes and shape the genetic predisposition to autoimmune conditions. J Exp Med.

[187] Raj T, Kuchroo M, Replogle JM, Raychaudhuri S, Stranger BE, De Jager PL (2013). Common risk alleles for inflammatory diseases are targets of recent positive selection. Am J Hum Genet.

[188] Zhernakova A, Elbers CC, Ferwerda B, Romanos J, Trynka G, Dubois PC (2010). Evolutionary and functional analysis of celiac risk loci reveals SH2B3 as a protective factor against bacterial infection. Am J Hum Genet.

[189] Uricchio LH, Zaitlen NA, Ye CJ, Witte JS, Hernandez RD (2016). Selection and explosive growth alter genetic architecture and hamper the detection of causal rare variants. Genome Res.

[190] Field Y, Boyle EA, Telis N, Gao Zu, Gaulton KJ, Golan D, et al. Detection of human adaptation during the past 2,000 years. Science. Oct 13 2016. Available from: https://www.ncbi.nlm.nih.gov/pubmed/27738015 [Epub ahead of print] 10.1126/science.aag0776 PMC5182071 27738015

[191] Prufer K, Racimo F, Patterson N, Jay F, Sankararaman S, Sawyer S (2014). The complete genome sequence of a Neanderthal from the Altai Mountains. Nature.

[192] Meyer M, Kircher M, Gansauge MT, Li H, Racimo F, Mallick S (2012). A high-coverage genome sequence from an archaic Denisovan individual. Science.

[193] Green RE, Krause J, Briggs AW, Maricic T, Stenzel U, Kircher M (2010). A draft sequence of the Neandertal genome. Science.

[194] Sankararaman S, Mallick S, Dannemann M, Prufer K, Kelso J, Paabo S (2014). The genomic landscape of Neanderthal ancestry in present-day humans. Nature.

[195] Reich D, Green RE, Kircher M, Krause J, Patterson N, Durand EY (2010). Genetic history of an archaic hominin group from Denisova Cave in Siberia. Nature.

[196] Reich D, Patterson N, Kircher M, Delfin F, Nandineni MR, Pugach I (2011). Denisova admixture and the first modern human dispersals into Southeast Asia and Oceania. Am J Hum Genet.

[197] Vernot B, Akey JM (2015). Complex history of admixture between modern humans and Neandertals. Am J Hum Genet.

[198] Vernot B, Akey JM (2014). Resurrecting surviving Neandertal lineages from modern human genomes. Science.

[199] Sankararaman S, Mallick S, Patterson N, Reich D (2016). The combined landscape of Denisovan and Neanderthal ancestry in present-day humans. Curr Biol.

[200] Simonti CN, Vernot B, Bastarache L, Bottinger E, Carrell DS, Chisholm RL (2016). The phenotypic legacy of admixture between modern humans and Neandertals. Science.

[201] Huerta-Sanchez E, Jin X, Asan, Bianba Z, Peter BM, Vinckenbosch N (2014). Altitude adaptation in Tibetans caused by introgression of Denisovan-like DNA. Nature.

[202] Abi-Rached L, Jobin MJ, Kulkarni S, McWhinnie A, Dalva K, Gragert L (2011). The shaping of modern human immune systems by multiregional admixture with archaic humans. Science.

[203] Mendez FL, Watkins JC, Hammer MF (2012). A haplotype at STAT2 Introgressed from neanderthals and serves as a candidate of positive selection in Papua New Guinea. Am J Hum Genet.

[204] Mendez FL, Watkins JC, Hammer MF (2012). Global genetic variation at OAS1 provides evidence of archaic admixture in Melanesian populations. Mol Biol Evol.

[205] Mendez FL, Watkins JC, Hammer MF (2013). Neandertal origin of genetic variation at the cluster of OAS immunity genes. Mol Biol Evol.

[206] Dannemann M, Andres AM, Kelso J (2016). Introgression of Neandertal- and Denisovan-like haplotypes contributes to adaptive variation in human Toll-like receptors. Am J Hum Genet.

